# Factors associated with diarrhea and acute respiratory infection in children under two years of age in rural Bangladesh

**DOI:** 10.1186/s12887-019-1738-6

**Published:** 2019-10-27

**Authors:** Md Barkat Ullah, Malay K. Mridha, Charles D. Arnold, Susana L. Matias, Md Showkat A. Khan, Zakia Siddiqui, Mokbul Hossain, Rina Rani Paul, Kathryn G. Dewey

**Affiliations:** 1 Department of Nutrition, University of California, One Shields Ave., Davis, CA 95616 USA; 2 Center for Non-communicable Disease and Nutrition, James P Grant School of Public Health, BRAC University, Dhaka, Bangladesh; 3 Department of Nutritional Sciences and Toxicology, University of California, 225 Morgan Hall, Berkeley, CA 94720 USA; 4 Health System and Population Studies Division, ICDDR,B, Dhaka, Bangladesh; 5 Care Bangladesh, Dhaka, Bangladesh

**Keywords:** Acute respiratory infection, Diarrhea, Child health, Child morbidity, Food insecurity, Maternal mental health

## Abstract

**Background:**

Diarrhea and acute respiratory infection (ARI) are major causes of child mortality. We aimed to identify risk factors associated with diarrhea and ARI among children under 2 years of age in rural northern Bangladesh.

**Method:**

We collected information on diarrhea and ARI in the previous 14 days and the previous 6 months at 6, 12, 18 and 24 months of age as part of a longitudinal, cluster randomized effectiveness trial, the Rang-Din Nutrition Study which enrolled 4011 pregnant women at ≤20 gestational weeks. Women and their children were followed up until 2 years postpartum. Information on household socioeconomic status, type of toilet, garbage disposal system, food insecurity, number of under-five children in the household, type of family, maternal characteristics and child characteristics was collected at baseline and/or at 6, 12, 18 and 24 months postpartum. Data on newborn health and feeding behaviors were collected within 72 h of delivery. Associations between potential risk factors and morbidity prevalence outcomes were assessed using logistic regression controlling for potential confounders.

**Results:**

Out of 3664 live born children, we collected information from ~ 3350 children at 6, 12, 18 and 24 months of age. Diarrhea in the previous 14 days, and in the previous 6 months, was associated with maternal depression score and food insecurity; diarrhea in the previous 6 months was also associated with family type (nuclear vs. joint). ARI in the previous 14 days was associated with maternal depression score, type of toilet and garbage disposal, household food insecurity and sex. Cough or nasal discharge in the past 6 months was associated with maternal depression score, type of toilet and garbage disposal, household food insecurity, sex and perceived overall physical condition of the infant after birth.

**Conclusion:**

Maternal depression and food insecurity appear to be important risk factors for diarrhea and respiratory infection among children under 2 years of age in this setting. These findings suggest that policies and programs that include strategies to address maternal mental health and household food insecurity may contribute to improved child health.

**Trial registration:**

The trial was registered with the US National Institutes of Health at ClinicalTrials.gov, # NCT01715038, with registration completed October 26, 2012.

## Introduction

Globally pneumonia and diarrhea are major causes of child mortality, accounting for 15.5 and 8.9%, respectively, of deaths among children under 5 years of age in 2015 [1]. The Millennium Development Goal (MDG) for reducing under-five mortality was not met at the global level [2], and the new goal, part of the Sustainable Development Goals, is to reduce under-five mortality to 25 per 1000 live births or less by 2030 [3]. In Bangladesh, the under-five mortality rate was 46 per 1000 live births in 2014 (Bangladesh Demographic and Health Survey 2014), and symptoms of acute respiratory infection (ARI) and diarrhea were present among 6 and 5%, respectively, of the under five children in the 2 weeks prior to the survey interview [4].

Incidence rates of respiratory infections [5–11] and diarrhea [11–17] are highest in the first 2 years of life and are related not only to child mortality but also to impaired physical growth and development of children [18]. Although many studies have been conducted to identify the factors associated with ARI and diarrhea in lower-income countries, most have included all children under 5 years of age, and have not focused on the first 2 years of life. Factors associated with both ARI and diarrhea among children under five include lower household Xsocio-economic status [5, 11, 13, 15, 19–21], poor sanitation [12, 15, 17, 20, 22], larger family size [6, 9, 12, 13, 17], living in a rural area [11, 14, 17, 23], younger mother [7, 11], low maternal education [11, 12, 15, 21, 22, 24], male child [10, 13, 15, 23], younger child [5–17], higher birth order of the child [9, 12, 22], suboptimal breastfeeding practices [9, 11, 24, 25], inadequate complementary feeding practices [20, 23, 24] and poor anthropometric status of the child [9, 11, 15, 20, 24]. Other factors associated with ARI include use of biomass fuel for cooking [5, 8, 24], low paternal education [24], and prematurity [24], and other factors associated with diarrhea include improper waste disposal [12, 17, 21, 22], improper drainage system [22, 26], unsafe drinking water [15, 26], maternal depression [27], improper hand washing [11, 15, 21–23] and lower birth order of the child [11].

There is little information on which of the above risk factors are most critical during the first 2 years of life. In addition, most of the above studies did not include household food insecurity or maternal mental health as potential risk factors in the analysis, even though these may be quite important in the etiology of infectious disease in young children. Food insecurity may be linked to greater risk of morbidity via inadequate nutrient intake, given the important role that several nutrients play in immune function [28–31]. Maternal mental health may affect child caregiving and thus also be related to risk of infection [27, 32]. We used data from a large intervention trial in rural Bangladesh, the Rang-Din Nutrition Study (RDNS) [33] to identify risk factors associated with ARI and diarrhea among children under 2 years of age.

## Methods

### Study setting

Data for these analyses come from a longitudinal, cluster-randomized effectiveness trial of nutritional supplements for pregnant and lactating women and their children through 2 years of age, conducted in 11 rural unions (lowest administrative unit of local government of Bangladesh) of the Badarganj and Chirirbandar subdistricts in northwest Bangladesh, as described previously [33]. It was implemented within the Community Health and Development Program (CHDP) operated by a local non-government organization (LAMB). Health services normally provided by the CHDP include maternity services at a safe delivery unit (SDU) in each union, regular home visits for antenatal, postnatal and child care by village health volunteers (VHV) and community health workers (CHW), and monthly educational sessions to promote maternal and child health. Clusters were defined as the work areas of the CHWs. Details regarding supplement composition, distribution scheme and key educational messages are provided elsewhere [33, 34]. The study protocol was approved by the institutional review boards of University of California, Davis (UCD); icddr,b; and LAMB. We obtained verbal consent from union representatives before beginning the study, and completed randomization of clusters before seeking individual participant’s written consent [33].

### Enrollment and data collection

The CHWs and VHVs identified pregnant women [33], and those potentially eligible for the RDNS were contacted at home by evaluation staff. Eligibility criteria included gestational age ≤ 20 weeks and no plans to move away during pregnancy or the following 3 years. All eligible women were invited to participate; those who consented were interviewed to collect baseline data as described elsewhere [33, 34].

Data collection was performed by 2 separate teams: the “SDU visit team,” which collected clinical and anthropometric data at the SDU, and the “home visit team,” which enrolled mothers and collected baseline and follow-up data at participants’ homes.

Baseline data on socioeconomic status, maternal characteristics, type of toilet, garbage disposal system, indoor air quality, number of under-five children in the household, family type and household food insecurity were collected at enrollment. For household socioeconomic status a set of 19 yes/no questions were asked about whether or not a household owned a particular item. These items included televisions, irrigation pumps, tables, bicycles, sewing machines, and other goods [33, 34]. We collected data on household food insecurity using the Household Food Insecurity Access Scale [35]. We assessed indoor air quality based on 5 questions about type of cooking facility, cooking location, type of fuel used for cooking, hours per day spent for cooking, and number of household members who smoked. Trained interviewers observed the type of toilet used and garbage disposal system.

Follow up during pregnancy and just after childbirth is described elsewhere [33]. At 6, 12, 18 and 24 months postpartum, mothers/caregivers were asked if their children had specific symptoms or illnesses: a) in the previous 2 weeks, and b) in the previous 6 months. Interviewers used standard procedures to help them understand the period of recall, and probed for each symptom or illness. For the 2-week recall, the symptom list included fever (any fever; high fever, defined as very hot to the touch), cough, nasal discharge fast breathing (faster than usual), difficulty in breathing, wheezing or grunting or whistling (any abnormal sound during breathing in or out), chest indrawing (not asked at 6 months interview), diarrhea (passage of abnormally loose or watery stool more than 3 times in a 24 h period), convulsion (shaking of hands, legs or body when ill), and lethargy/drowsiness (too weak to speak, move or play normally). If the mother/caregiver reported “yes” for any of the symptoms, she was then asked how many days in the last 2 weeks the child had that symptom. If the mother/caregiver mentioned two or more symptoms in the last 2 weeks, she was also asked which of those symptoms were present at the same time. For the 6-month recall, the mother/caregiver was asked how many episodes of three common illnesses or sets of symptoms her child experienced: 1) cough or nasal discharge 2) cough or nasal discharge accompanied by fast or difficult breathing (this question was nested within the first question, so that if the answer to the first question was one or more, the second question was asked), and 3) diarrhea. Feeding history of the children was collected from their mothers/caregivers at 6, 12, 18 and 24 months by 24 h recall. To the extent possible, data collectors were kept blind to group assignment, although those conducting home visits might have seen supplements in the home. Quality-control procedures [33] included having supervisors re-interview at least 10% of randomly selected participants.

### Outcome definitions

Primary outcomes based on the 2-week recalls (at 6, 12, 18 and 24 months) are the percentage of children with any occurrence of: a) diarrhea (defined as stated above), b) acute upper respiratory infection (AURI), and c) acute lower respiratory infection (ALRI) during the previous 2 weeks. AURI is defined as caregiver report of cough and nasal discharge on the same day(s) but no difficulty in breathing, rapid breathing, wheezing/grunting/whistling, chest indrawing, convulsion or lethargy during those days. ALRI is defined as caregiver report of cough together with at least one of the following symptoms on the same day(s): difficulty in breathing, rapid breathing, wheezing/grunting/whistling or chest indrawing. Secondary outcomes based on the 6-month recalls are the percentage of children with any occurrence of a) cough or nasal discharge, b) cough or nasal discharge accompanied by fast or difficult breathing, and c) diarrhea in the previous 6 months.

### Predictor variables

The potential risk factors included in these analyses were based on data collected at various time points (Additional file 1: Table S1) on household socioeconomic status (asset index), type of toilet, garbage disposal system, food insecurity, number of under-five children in the household, type of family (nuclear or extended), maternal characteristics (education and depression), and child characteristics (sex, low birth weight (< 2500 g), prematurity (< 37 weeks gestation), overall perceived physical condition of the newborn soon after birth (healthy or not healthy), stunting (length-for-age Z-score [LAZ] < − 2), and dietary diversity). We excluded indoor air quality from these analyses because more than 99% of households used bio-mass fuel for cooking and cooked on an open fire, yielding little variability in this potential predictor.

We used principal components analysis (PCA) to calculate a household asset index from the set of 19 yes/no questions about whether or not a household owned a particular item; higher values represented higher socioeconomic status. Toilet facility was categorized into best (sanitary and water seal latrines), intermediate (pit latrine with slab and water seal) and worst (pit latrine without water seal, hanging/open latrine or no latrine). Garbage disposal system was categorized into better (burned or dumped in a designated place) and worse (no specific place to dump or dumped in open space or dumped in pond/river/canal). Household food insecurity was categorized into four levels of household food insecurity (severe, moderate, mild, and none) [33]. We defined maternal depression based on the top decile of maternal depression scores in the study population at each time point (baseline, 6 and 24 months postpartum) using a 10 point scale modified Bengali version of the Edinburgh Postpartum Depression Scale (EPDS) [36] screening tool. We used the top decile of maternal depression scores instead of using a standard cut-off because the percentage of women with scores above the standard cut-off was very low. At baseline, 6 months and 24 months the top decile cutoffs were > 7, > 5 and > 5, respectively. Child minimum dietary diversity (MDD) was defined as reported consumption during the previous 24 h of foods from four or more food groups out of the seven food groups as defined by the World Health Organization for this indicator [37].

### Statistical analyses

Maternal and child characteristics were examined using frequencies and percentages for categorical data and either mean and SD or median and IQR or quartiles for continuous variables. To examine the variables related to the dichotomous morbidity outcomes, we used logistic regression and all models were longitudinal, which means they included all time points of data collected for a child. To account for the cluster design of the trial and repeated measurements on the same child we used robust sandwich variance estimation with clusters as the independent unit. All models controlled for intervention group assignment and age category of data collection. If data were missing at a time point, then that time point was not included for that child. For models of morbidity outcomes based on the 2-week recalls, predictor variables were based on concurrent or the most recently collected information (Additional file 1: Table S2). The same predictor variables were used for analyzing all morbidity outcomes.

For models of morbidity outcomes based on the 6-month recalls, predictor variables were based on information collected at the beginning of each 6-month period or as close to that time point as possible (Additional file 1: Table S3). We first assessed bivariate associations and used odds ratios (ORs) and descriptive statistics to understand the direction of the relationships. Then in separate models we assessed the interaction between predictor and age category to determine whether the association between predictor and outcome varied by age, and created figures to visualize significant interactions. We controlled for multiple hypothesis testing by correcting bivariate and interaction *p*-values using Benjamini-Hochberg multiple hypothesis correction [38]. Predictors significant in bivariate analyses were included in a multivariable model to assess whether they retained significance when controlling for other predictor variables. Collinearity was first assessed using a variance inflation factor cutoff of 5 and if necessary, a single predictor was selected from predictors representing the same construct.

## Results

A total of 3664 live births occurred between January 15, 2012 and May 5, 2013, as described elsewhere [33]. There were 30 twin deliveries (including stillbirth) and one twin from each pair was randomly selected for analysis. The numbers of children for whom data were collected at each time point are shown in the Additional file 2: Figure S1; at 24 months of age, more than 90% of the live born children remained in the study. Socioeconomic characteristics of the mother-infant dyads who were lost to follow up were compared with those of the dyads who were included in this analysis. Dyads lost to follow-up differed in several ways including greater maternal age, lower maternal height, lower maternal education, shorter duration of gestation, more likely to be a nuclear family, lower household assets, greater food insecurity, higher percentage of preterm delivery, lower birth weight and greater likelihood of newborn stunting, compared to those who remained in the analysis (Table 1).

**Table 1:** Socioeconomic, maternal and child characteristics of the mother-infant dyads in the analyses, compared to those who were lost to follow up

	Not lost to follow-up (Mean ± SD)	Lost to follow-up (Mean ± SD)	*P*-value
Maternal characteristics	*n* = 3530	*n* = 481	
Age, (y)	21.9 ± 4.9	22.4 ± 5.6	0.023
Height (cm)	150.6 ± 5.4	149.9 ± 5.3	0.005
BMI (kg/m^2^)	20.0 ± 2.7	19.9 ± 2.7	0.752
Years of education	6.3 ± 3.2	5.6 ± 3.4	< 0.001
Nulliparous, n (%)	1404 (39.8)	187 (39.4)	0.886
Maternal depression score in the top decile (%)	9.6	10.8	0.190
Gestational age at enrolment, (d)	92.0 ± 26.9	91.2 ± 24.4	0.505
Gestational age at birth (wk)	39.4 ± 2.2	37.9 ± 3.7	< 0.001
Type of toilet (%)			0.231
Best	13.1	10.4	
Intermediate	25.6	25.8	
Worse	61.3	63.8	
Better garbage disposal (%)	74.7	74.2	0.855
Number of children < 5 y in the household	0.43 ± 0.60	0.39 ± 0.60	0.319
Nuclear family (%)	73.7	79.8	0.007
Household asset index	0.04 ± 2.27	−0.33 ± 2.12	0.003
Household food insecurity, n, (%)			0.039
Food secure	1696 (48.0)	205 (42.6)	
Mildly food insecure	506 (14.3)	75 (15.6)	
Moderately food insecure	1020 (28.9)	143 (29.7)	
Severely food insecure	308 (8.7)	58 (12.1)	
Child characteristics	*n* = 3517	*n* = 228	
Sex, male, n, (%)	1770 (50.3)	110 (48.2)	0.589
Stunting at birth (%)	21.0	54.9	< 0.001
Perceived as healthy (%)	91.7	54.2	< 0.001
Prematurity (%)	13.1	39.4	< 0.001
Birth weight (g)	2610 ± 400	2160 ± 600	< 0.001

In this sample, less than 13.1% of the households had a flush or water seal toilet, while 74.7% had a garbage disposal system categorized as “better”. At baseline 8.7, 28.9, 14.3 and 48.0% of households were categorized as having severe, moderate, mild and no food insecurity, respectively. Average family size was ~ 4.6, about 37.5% of the households had at least one child under 5 years of age at baseline and most (73.7%) were nuclear families. Mean maternal age was 21.9 years, on average they attended 6.3 years in school, 39.8% of the women were nulliparous and their average gestational age at enrollment was 13 weeks. Half of the infants were male. Average birth weight was 2610 g, prevalence of low birth weight was 29.5, and 13.1% were born preterm. Nearly all (91.7%) of the newborns were rated as “healthy” based on the overall condition of the baby soon after birth. The prevalence of stunting was 24.0, 28.3, 34.7 and 40.6% at 6, 12, 18 and 24 months, respectively. MDD was 2.9, 53.2, 66.3 and 74.4% at 6, 12, 18 and 24 months, respectively.

### Factors associated with prevalence of diarrhea

#### Previous 2 weeks

The percentage of children with reported diarrhea in the previous 2 weeks was 6.8, 7.8, 7.5 and 3.4% at 6, 12, 18 and 24 months, respectively. Asset index, maternal education, maternal depression score in the top decile, food insecurity and family type (nuclear vs joint) were significantly associated with the prevalence of diarrhea in the previous 2 weeks in bivariate analysis, whereas type of toilet, garbage disposal system, minimum dietary diversity, number of under-five children in the household, sex of the child, low birth weight, prematurity, stunting and rating of the overall condition of the infant soon after birth were not associated with diarrhea prevalence in the previous 2 weeks (data not shown).

In multivariable analysis (Table 2, which includes only the predictors that were significantly related to at least one outcome), diarrhea was more likely among children of mothers scoring in the top decile of the depression screening tool [AOR (95% CI), 1.58 (1.31, 1.90)] compared to those scoring lower, and among children in households with moderate [AOR (95% CI), 1.23 (1.01, 1.50)] or severe [AOR (95% CI), 1.33 (1.00, 1.75)] food insecurity compared to those in households with no food insecurity. Diarrhea was less likely among children in joint families [AOR (95% CI), 0.72 (0.61, 0.85)] compared to those in nuclear families.

**Table 2:** Association of diarrhea and respiratory infections in the previous 2 weeks with potential risk factors

Variables	Observations with diarrhea		Observations with ALRI		Observations with AURI	
	%	AOR (95% CI)	%	AOR (95% CI)	%	AOR (95% CI)
Maternal depression score in the top decile						
Yes	9.7	**1.58 (1.31, 1.90)**	17.7	**1.29 (1.10, 1.51)**	30.6	–
No	6.0	–	12.7	–	30.3	–
Type of toilet						
Best	5.3	–	11.1	–	29.4	–
Intermediate	6.7	–	16.5	**1.31 (1.10, 1.56)**	28.4	–
Worst	6.7	–	13.0	0.94 (0.81, 1.09)	31.2	–
Garbage disposal						
Best	6.5	–	12.7	–	30.2	–
Worst	5.5	–	16.5	**1.30 (1.13, 1.49)**	29.8	–
Food insecurity						
Food secure	5.7		11.5		29.2	
Mild food insecurity	7.4	1.17 (0.92, 1.50)	14.5	**1.21 (1.01, 1.45)**	29.4	1.01 (0.89, 1.14)
Moderate food insecurity	7.9	**1.23 (1.01, 1.50)**	17.8	**1.59 (1.42, 1.78)**	32.8	**1.18 (1.07, 1.30)**
Severe food insecurity	8.1	**1.33 (1.00, 1.75)**	20.0	**1.95 (1.55, 2.45)**	34.2	**1.24 (1.02, 1.51)**
Family type						
Nuclear	6.6		13.1		29.9	–
Joint family	5.0	**0.72 (0.61, 0.85)**	14.1	–	30.9	–
Sex of the child						
Male	6.1	–	14.8	–	29.7	–
Female	6.7	–	11.8	**0.76 (0.68, 0.85)**	30.4	–

Multivariable adjusted odds ratio and 95% confidence interval for occurrence of diarrhea, ALRI and AURI in the previous 2 weeks before 6, 12, 18 and 24 months of age. Models are longitudinal and control for time point of data collection. First category for all predictors has been considered as the reference category. Dashes signify non-significant relationships and reference categories. Only the predictors that were significantly associated with at least one outcome are shown. AORs in boldface are statistically significant at *p* < 0.05; AORs not in boldface are not significant but are provided for completeness for all categories of those predictor variables

#### Previous 6 months

The percentage of children with any occurrence of reported diarrhea in the previous 6 months was 20.1, 28.8, 28.8 and 16.9% at 6, 12, 18 and 24 months, respectively. Maternal depression score and food insecurity were significantly associated with prevalence of diarrhea in the last 6 months in bivariate analysis, whereas asset index, maternal education, type of toilet, garbage disposal system, dietary diversity, number of under-five children in the household, type of family, sex of the child, low birth weight, prematurity, stunting and perceived overall condition of the infant soon after birth were not associated with diarrhea prevalence in the previous 6 months (data not shown).

In multivariable analysis (Table 3, which includes only the predictors that were significantly related to at least one outcome), diarrhea was more likely among children of mothers scoring in the top decile of the depression screening tool [AOR (95% CI), 1.43 (1.28, 1.60)] compared to those scoring lower, and among children in households with mild [AOR (95% CI), 1.15 (1.02, 1.30)], moderate [AOR (95% CI), 1.26 (1.15, 1.39)] or severe [AOR (95% CI), 1.47 (1.22, 1.77)] food insecurity compared to those in households with no food insecurity.

**Table 3:** Association of diarrhea and respiratory infections in the previous 6 months with potential risk factors

Variables	Observations with diarrhea		Observations with cough or nasal discharge		Observations with cough or nasal discharge accompanied with fast breathing	
	%	AOR (95% CI)	%	AOR (95% CI)	%	AOR (95% CI)
Maternal depression score in the top decile						
Yes	31.0	**1.43 (1.28, 1.60)**	92.0	**1.44 (1.14, 1.83)**	28.1	**1.38 (1.18, 1.62)**
No	22.7	–	88.4	–	22.0	–
Type of toilet						
Best	21.4	–	85.4	–	19.1	–
Intermediate	23.9	–	90.4	**1.26 (1.04, 1.53)**	26.0	**1.18 (1.02, 1.36)**
Worst	24.4	–	89.8	1.10 (0.95, 1.27)	22.9	1.00 (0.88, 1.14)
Garbage disposal						
Best	23.6	–	88.6	–	22.0	–
Worst	23.8	–	90.0	–	26.0	**1.17 (1.03, 1.33)**
Food insecurity						
Food secure	21.6		87.0		19.4	
Mild food insecurity	26.7	**1.15 (1.02, 1.30)**	92.8	**1.43 (1.14, 1.79)**	28.8	1.11 (0.96, 1.28)
Moderate food insecurity	28.1	**1.26 (1.15, 1.39)**	92.8	**1.31 (1.11, 1.54)**	29.0	**1.33 (1.21, 1.45)**
Severe food insecurity	31.0	**1.47 (1.22, 1.77)**	93.3	**1.68 (1.17, 2.40)**	33.5	**1.62 (1.33, 1.98)**
Sex of the child						
Male	24.3	–	89.7	–	24.4	
Female	23.0	–	87.9	**0.82 (0.72, 0.94)**	20.9	**0.81 (0.73, 0.89)**
Maternal perception of infant condition at birth						
Healthy	23.5	–	88.6	–	22.5	–
Not healthy	25.4	–	91.9	**1.49 (1.16, 1.91)**	25.0	–

Multivariable adjusted odds ratio and 95% confidence interval for occurrence of any episode of diarrhea, cough or nasal discharge, and cough or nasal discharge accompanied with fast breathing or difficulty in breathing in the last 6 months before 6, 12, 18 and 24 months of age. Models are longitudinal and control for time point of data collection. First category for all predictors has been considered as the reference category. Dashes signify non-significant relationships and reference categories. Only the predictors that were significantly associated with at least one outcome are shown. AORs in boldface are statistically significant at *p* < 0.05; AORs not in boldface are not significant but are provided for completeness for all categories of those predictor variables

### Factors associated with respiratory infections

#### ALRI in the previous 2 weeks

The percentage of children with reported ALRI in the previous 2 weeks was 17.6, 15.2, 11.6 and 8.8% at 6, 12, 18 and 24 months, respectively. Maternal depression score, type of toilet, garbage disposal system, food insecurity and sex of the child were significantly associated in bivariate analyses with prevalence of ALRI, whereas asset index, maternal education, dietary diversity, type of family, number of under-five children in the household, low birth weight, prematurity, stunting and perceived overall condition of the infant soon after birth were not associated with ALRI prevalence (data not shown).

In multivariable analysis (Table 2), ALRI was more likely among children of mothers scoring in the top decile of the depression screening tool [AOR (95% CI), 1.29 (1.10, 1.51)] compared to those scoring lower, among children in households with “intermediate” quality toilets [AOR (95% CI), 1.31 (1.10, 1.56)] compared to those in households with “best” quality toilets, among children in households with “worse” garbage disposal systems [AOR (95% CI), 1.30 (1.13, 1.49)] compared to those in households with “better” garbage disposal systems, and among children in households with mild [AOR (95% CI), 1.21 (1.01, 1.45)], moderate [AOR (95% CI), 1.59 (1.42, 1.78)] or severe [AOR (95% CI), 1.96 (1.55, 2.45)] food insecurity compared to those in the households with no food insecurity. ALRI was less likely among female children [AOR (95% CI), 0.76 (0.68, 0.85)] compared to male children.

#### AURI in the previous 2 weeks

The percentage of children with reported AURI in the previous 2 weeks was 30.6, 30.1, 30.0 and 29.6% at 6, 12, 18 and 24 months, respectively. Only food insecurity was significantly associated with prevalence of AURI in bivariate analysis, whereas asset index, maternal education, maternal depression score, type of toilet, garbage disposal system, dietary diversity, type of family, number of under-five children in the household, sex of the child, low birth weight, prematurity, stunting and perceived overall condition of the infant soon after birth were not associated with AURI prevalence (data not shown).

In multivariable analysis (Table 2), AURI was more likely among children in households with moderate [AOR (95% CI), 1.18 (1.07, 1.30)] or severe [AOR (95% CI), 1.24 (1.02, 1.51)] food insecurity compared to those in households with no food insecurity.

#### Cough or nasal discharge in the previous 6 months

The percentage of children with cough or nasal discharge in the previous 6 months was 97.8, 92.9, 84.4 and 80.0% at 6, 12, 18 and 24 months, respectively. Maternal depression score, type of toilet, food insecurity, sex of the child, prematurity and perceived overall condition of the infant soon after birth were significantly associated with prevalence of cough or nasal discharge in the previous 6 months in bivariate analysis, whereas asset index, maternal education, garbage disposal system, dietary diversity, type of family, number of under-five children in the household, low birth weight and stunting were not associated with cough or nasal discharge prevalence (data not shown).

In multivariable analysis (Table 3), cough or nasal discharge was more likely among children of mothers scoring in the top decile of the depression screening tool [AOR (95% CI), 1.44 (1.14, 1.83)] compared to those scoring lower, among children in households with “intermediate” quality toilets [AOR (95% CI), 1.26 (1.04, 1.53)] compared to those in households with “best” quality toilets, among children in households with mild [AOR (95% CI), 1.43 (1.14, 1.79)], moderate [AOR (95% CI), 1.31 (1.11, 1.54)] or severe [AOR (95% CI), 1.68 (1.17, 2.40)] food insecurity compared to those in households with no food insecurity, and among children rated as “not healthy” soon after birth [AOR (95% CI), 1.49 (1.16, 1.91)] compared to those rated as “healthy”. Cough or nasal discharge was less likely among female children [AOR (95% CI), 0.82 (0.72, 0.94)] compared to male children.

#### Cough or nasal discharge accompanied by fast or difficult breathing in the previous 6 months

The percentage of children with cough or nasal discharge accompanied by fast or difficult breathing in the previous 6 months was 38.6, 24.0, 15.6 and 12.3% at 6, 12, 18 and 24 months, respectively. Maternal depression score, type of toilet, garbage disposal system, food insecurity and sex of the child were significantly associated with prevalence of this outcome in bivariate analysis, whereas asset index, maternal education, dietary diversity, type of family, number of under-five children in the household, low birth weight, prematurity, stunting and perceived overall condition of the infant soon after birth were not associated with this outcome (data not shown).

In multivariable analysis (Table 3), cough or nasal discharge accompanied by fast or difficult breathing was more likely among children of mothers scoring in the top decile of the maternal depression screening tool [AOR (95% CI), 1.38 (1.18, 1.62)] compared to those scoring lower, among children in households with “intermediate” quality toilets [AOR (95% CI), 1.18 (1.02, 1.36)] compared to those in households with “best” quality toilets, among children in households with better garbage disposal systems (compared to worse) [AOR (95% CI), 1.17 (1.03, 1.33)], and among children in households with moderate [AOR (95% CI), 1.33 (1.21, 1.45)] or severe [AOR (95% CI), 1.62 (1.33, 1.98)] food insecurity compared to those in households with no food insecurity. Cough or common cold accompanied by fast or difficult breathing was less likely among female children [AOR (95% CI), 0.81 (0.73, 0.89)] compared to male children.

## Discussion

This study aimed to identify risk factors associated with diarrhea and respiratory infection in children under 2 years of age in rural Bangladesh. Controlling for other factors, we found that scoring in the top decile on the maternal depression screening tool and household food insecurity were associated with higher likelihood of reported diarrhea and respiratory infections in both short (previous 2 weeks) and long term (previous 6 months) recall periods. In addition, male children were more likely than female children to experience respiratory infections, children living in households with better quality toilets or garbage disposal systems had lower prevalence of respiratory infections, and those living in joint (vs. nuclear) families had lower prevalence of diarrhea. Other potential risk factors, such as number of children under five in the household, low birth weight, stunting and dietary diversity, were not associated with these morbidity outcomes in either bivariate or multivariable analyses in this population.

The positive association between a higher maternal depression score and reported diarrhea is consistent with findings from another cohort study in Pakistan, in which a greater number of diarrheal episodes were reported from birth to 12 months among infants of mothers who were identified as having prenatal clinical depression compared to those of psychologically well mothers [RR (95%CI), 2.4 (1.7; 3.3)] [27]. A possible explanation for this relationship is compromised maternal ability to provide proper care for her child. In the Pakistan study, infants of the mothers who were clinically depressed during late pregnancy were less likely to be fully immunized at 12 months [27]. We also found a positive association between higher maternal depression score and respiratory infection, whereas this relationship was not observed in the Pakistan study [27]. Other investigators have demonstrated that depressed mothers tend to have negative views about their parenting ability [32] and they respond more slowly to their children [39], which could negatively influence the physical wellbeing of their children. In Sweden, the rate of hospital admission and mortality was higher among preschool children of mentally ill mothers [40]. In our study, recall bias or reverse causality cannot be excluded, e.g. a depressed mother may be more likely to view her child as sickly and be more likely to recall illness episodes.

We were unable to locate other studies that have examined the relationship between household food insecurity and diarrhea or respiratory infections. However, food insecurity has been associated with poor health outcomes of children in low income families in the U.S. [41]. Food insecurity may reduce intake of nutrients that play a role in immune function [28–31, 42–44], which could explain the associations we observed between food insecurity and diarrheal and respiratory infection. More surprising was our finding that children living in joint families had less reported diarrhea in the previous 2 weeks compared to those living in nuclear families. Other studies have shown that children in larger families are more vulnerable to diarrhea [12, 13, 17], which may be related to quality of care and hygiene. It is possible that the joint families in our study area included more adult caregivers, compared to nuclear families, which might have enhanced the quality of care.

Our finding of greater reported prevalence of respiratory infections among boys than girls is consistent with a case-control study in Thailand [10], a cohort study in Bangladesh [45], and a systematic review of studies on hospital admission for severe acute lower respiratory infection among children under five [46]. Higher risk of infection in male children could be related to stress related thymic atrophy in male but not female infants, a phenomenon found in a study in Bangladesh [47], as smaller thymic size is associated with greater risk of infection [48]. Another possibility is that the suppression of immune response by testosterone [49, 50] could play a role, as a peak in sex hormones is observed during infancy, often referred as mini puberty [50]. The observed association is consistent with the well-known higher infant mortality rate among males compared to females [51–53]. The prevalences of all three of the morbidity outcomes based on 6-month recalls were slightly higher among the children who were rated “not healthy” soon after birth, but only the difference in prevalence of AURI was statistically significant. This suggests that the overall perceived condition of the newborn may have implications for subsequent health outcomes. Further research is needed to identify ways to reduce the risk of infection in infants who are perceived as more vulnerable.

We did not find any relationship of low birth weight or stunting at the beginning of each age interval with subsequent diarrhea or respiratory infection. A cross-sectional study of children under five in northern Bangladesh also found no relationship between stunting and respiratory infection [6]. However, low birth weight was associated with greater risk of respiratory infection among infants in a prospective cohort study in Turkey [24] and among children under five in developing countries in a 1991 review on the epidemiology of ARI [9]. It is possible that widespread access to primary health care in our study area reduced potential disparities in vulnerability of children to the types of infections reported herein.

Strengths of this study include the following: 1) large sample size; 2) all data including morbidity and anthropometric data were collected by a well-trained and standardized team; 3) newborn data were collected within 72 h of birth; and 4) there was a low rate of loss to follow-up at all four time points. The key limitations are that all of the morbidity outcomes were based on maternal or caregiver recall of symptoms, with a recall period of 2 weeks for some of the outcomes and 6 months for others. Other limitations are that a) information for some predictors was not available at all time points, b) generalizability of our findings is limited due to greater loss to follow-up of infants who were born preterm or with low birth weight and differences in maternal and child characteristics of this sample of children compared to those of the original study sample enrolled, c) the definition of diarrhea in our study was more restrictive than that of other studies, i.e., > 3 (rather than > 3) abnormally loose or watery stools in a 24 h period, and d) the use of a non-standard definition for maternal depression because the percentage of women with scores above the standard cut-off was very low.

## Conclusion

We conclude that a higher maternal depression score and food insecurity appear to be important risk factors for diarrhea and respiratory infection among children under 2 years of age in this setting. These findings suggest that policies and programs that include strategies to address maternal mental health and household food insecurity may contribute to improved child health. Interventions based on the principles of cognitive behavior therapy, which can be delivered by primary health workers as demonstrated in Pakistan [54], may be feasible options to tackle the burden of maternal depression in this population. Experience-based household food security scales could be used to identify households with food insecurity and target them for interventions such as food rations, food for work, or conditional cash transfer programs. Evaluations of such interventions should include child health among the outcomes assessed when documenting effectiveness and cost-effectiveness.

## Supplementary information

**Additional file 1: Table S1.** Time points of data collection for predictor variables. **Table S2.** Predictor variables used at each time point in the analyses of associations with the morbidity outcomes for the previous 2 weeks. **Table S3.** Predictor variables used at each time point in the analyses of associations with the morbidity outcomes for the previous 6 months.

**Additional file 2: Figure S1.** Study flow chart. ^1^366 gestational age > 140 days; 22 planned to leave the study site; 8 refused to consent and 3 husbands refused to consent. ^2^Most of these deaths occurred at < 14 d postpartum: 14 IFA-Control, 15 IFA-MNP, 15 IFA-LNS, and 17 LNS-LNS. IFA-Control, women received iron and folic acid supplement during pregnancy and the first 3 months postpartum and children did not receive supplements; IFA-LNS, women received iron and folic acid during pregnancy and the first 3 months postpartum and children received lipid-based nutrient supplements from 6 to 24 months of age; IFA-MNP, women received iron and folic acid during pregnancy and the first 3 months postpartum and children received micronutrient powder from 6 to 24 months of age; LNS-LNS, women and children received lipid-based nutrient supplements. “No show” refers to participants for whom data collection was not completed at that time point, primarily due to travel out of the study area or refusal because of illness or other reasons.

## Data Availability

The datasets generated and/or analyzed during the current study are not yet publicly available but are available from the corresponding author on reasonable request.

## Abbreviations

ALRI: Acute lower respiratory infection
ARI: Acute respiratory infection
AURI: Acute upper respiratory infection
CHDP: Community Health and Development Program
CHW: Community health worker
EPDS: Edinburgh Postpartum depression scale
FANTA: Food and Nutrition Technical Assistance project
IFA: Iron and folic acid
LAMB: Lutheran Aid for Medicine in Bangladesh (a non-government organization in Dinajpur Bangladesh)
LNS: Lipid-based nutrient supplement
LNS-C: Lipid-based nutrient supplement for children
LNS-PL: Lipid-based nutrient supplement for pregnant and lactating women
MDD: Minimum dietary diversity
MNP: Micronutrient powder
PCA: Principal component analysis
RDNS: Rang-Din Nutrition Study
SDU: Safe delivery unit
UCD: University of California, Davis
VHV: Village health volunteer
