# Supplementary material for: Factors associated with diarrhea and acute respiratory infection in children under two years of age in rural Bangladesh
Source: BMC Pediatr. 2019 Oct 27;19:386. doi: 10.1186/s12887-019-1738-6 (PMC6815354; doi:10.1186/s12887-019-1738-6)
Supplement: Supplementary file 1 — Additional file 1: Table S1. Time points of data collection for predictor variables. Table S2. Predictor variables used at each time point in the analyses of associations with the morbidity outcomes for the previous 2 weeks. Table S3. Predictor variables used at each time point in the analyses of associations with the morbidity outcomes for the previous 6 months. [file 12887_2019_1738_MOESM1_ESM.docx]

**Table S1: Time points of data collection for predictor variables.**

|  | | **Time of data collection** | | | | | |
| --- | --- | --- | --- | --- | --- | --- | --- |
| **Variable** | | **Baseline** | **Birth visit** | **6 mo** | **12 mo** | **18 mo** | **24 mo** |
| Asset index | | x | - | x | - | x | x |
| Type of toilet | | x | - | x | - | x | x |
| Garbage disposal system | | x | - | x | - | x | x |
| Food insecurity | | x | - | x | x | x | x |
| Number of under-five children in the household | | x | - | x | - | x | x |
| Family type | | x | - | x | - | x | x |
| Maternal education | | x | - |  | - | - | - |
| Maternal depression score in the top decile | | x | - | x | - | - | x |
| Child characteristics | |  |  |  |  |  |  |
|  | Sex | - | x | - | - | - | - |
|  | Low birth weight (<2500 g) | - | x | - | - | - | - |
|  | Prematurity (<37 gestational weeks) | - | x | - | - | - | - |
|  | Overall perceived physical condition of the newborn at birth | - | x | - | - | - | - |
|  | Stunting (LAZ < -2) | - | x | x | x | x | x |
|  | Minimum dietary diversity score | - | - | x | x | x | x |

**Table S2:** **Predictor variables used at each time point in the analyses of associations with the morbidity outcomes for the previous two weeks.**

| **Variable** | | **Morbidity during the previous two weeks of** | | | |
| --- | --- | --- | --- | --- | --- |
|  |  | **6 mo** | **12 mo** | **18 mo** | **24 mo** |
| Asset index | | 6 mo | 6 mo | 18 mo | 24 mo |
| Type of toilet | | 6 mo | 6 mo | 18 mo | 24 mo |
| Garbage disposal system | | 6 mo | 6 mo | 18 mo | 24 mo |
| Food insecurity | | 6 mo | 12 mo | 18 mo | 24 mo |
| Number of <5 children in the household | | 6 mo | 6 mo | 18 mo | 24 mo |
| Family type | | 6 mo | 6 mo | 18 mo | 24 mo |
| Maternal education | | Baseline | Baseline | Baseline | Baseline |
| Maternal depression score in the top decile | | 6 mo | 6 mo | 6 mo | 24 mo |
| Child characteristics | |  |  |  |  |
|  | Sex | At birth | At birth | At birth | At birth |
|  | Low birth weight (<2500 g) | At birth | At birth | At birth | At birth |
|  | Prematurity (< 37 gestational weeks) | At birth | At birth | At birth | At birth |
|  | Overall perceived physical condition of the newborn at birth | At birth | At birth | At birth | At birth |
|  | Stunting (LAZ < -2) | 6 mo | 12 mo | 18 mo | 24 mo |
|  | Minimum dietary diversity score | - | 12 mo | 18 mo | 24 mo |

**Table S3: Predictor variables used at each time point in the analyses of associations with the morbidity outcomes for the previous six months.**

|  | | **Morbidity during 6 mo interval** | | | |  |
| --- | --- | --- | --- | --- | --- | --- |
| **Variable** | | **0-6 mo** | **6-12 mo** | **12-18 mo** | **18-24 mo** | |
| Asset index | | Baseline | 6 mo | 18 mo | 18 mo | |
| Type of toilet | | Baseline | 6 mo | 18 mo | 18 mo | |
| Garbage disposal system | | Baseline | 6 mo | 18 mo | 18 mo | |
| Food insecurity | | Baseline | 6 mo | 12 mo | 18 mo | |
| Number of <5y children in the household | | Baseline | 6 mo | 18 mo | 18 mo | |
| Family type | | Baseline | 6 mo | 18 mo | 18 mo | |
| Maternal education | | Baseline | Baseline | Baseline | Baseline | |
| Maternal depression score in the top decile | | Baseline | 6 mo | 6 mo | 24 mo | |
| Child characteristics | |  |  |  |  | |
|  | Sex | At birth | At birth | At birth | At birth | |
|  | Low birth weight (<2500 g) | At birth | At birth | At birth | At birth | |
|  | Prematurity (<37 gestational weeks) | At birth | At birth | At birth | At birth | |
|  | Overall perceived physical condition of the newborn at birth | At birth | At birth | At birth | At birth | |
|  | Stunting (LAZ < -2) | At birth | 6 mo | 12 mo | 18 mo | |
|  | Minimum dietary diversity score |  | 6 mo | 12 mo | 18 mo | |
