# Supplementary figures and images for: Factors associated with diarrhea and acute respiratory infection in children under two years of age in rural Bangladesh
Source: BMC Pediatr. 2019 Oct 27;19:386. doi: 10.1186/s12887-019-1738-6 (PMC6815354; doi:10.1186/s12887-019-1738-6)

## Study flow chart

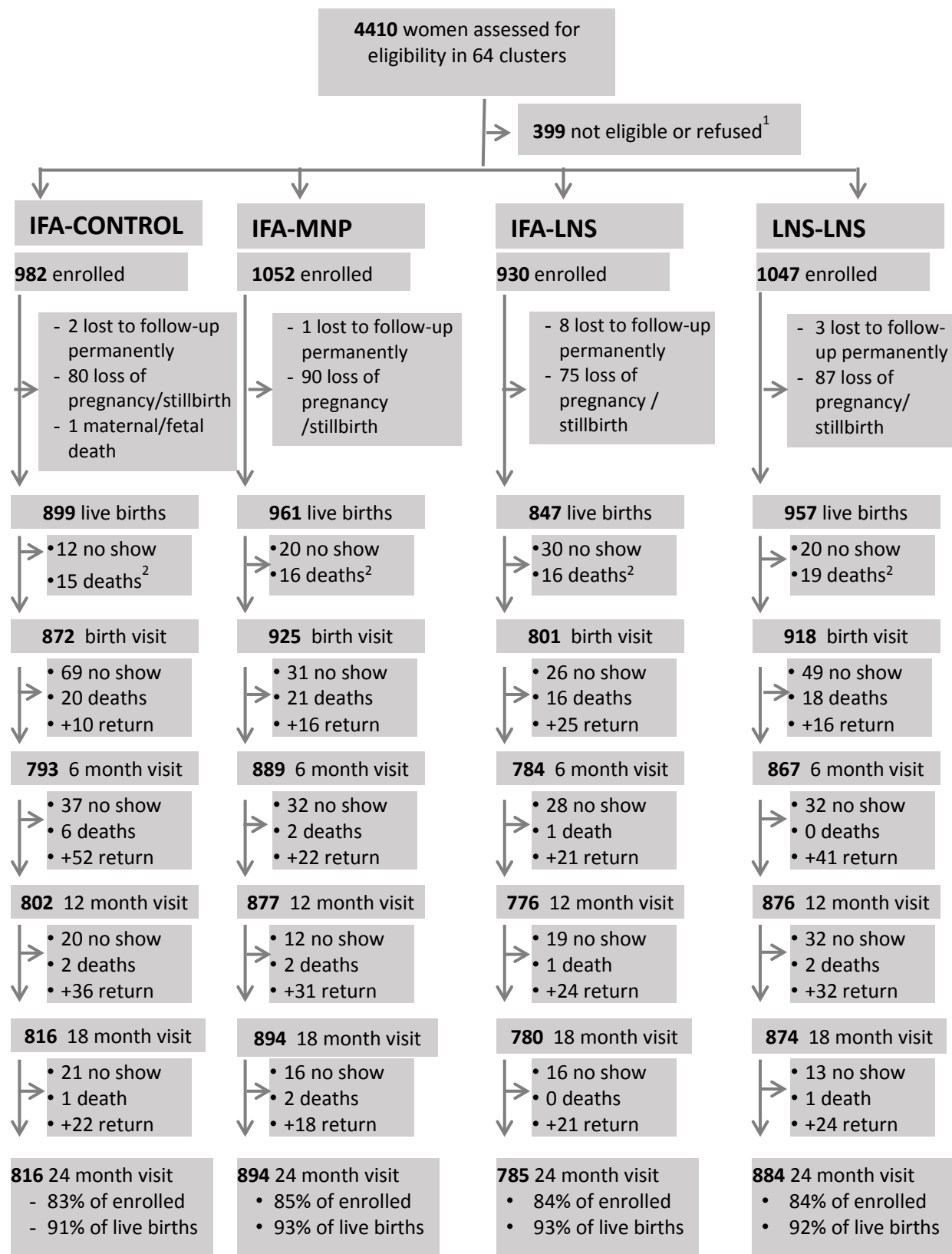

Supplement: Supplementary file 2 — Additional file 2: Figure S1. Study flow chart. 1366 gestational age > 140 days; 22 planned to leave the study site; 8 refused to consent and 3 husbands refused to consent. 2Most of these deaths occurred at < 14 d postpartum: 14 IFA-Control, 15 IFA-MNP, 15 IFA-LNS, and 17 LNS-LNS. IFA-Control, women received iron and folic acid supplement during pregnancy and the first 3 months postpartum and children did not receive supplements; IFA-LNS, women received iron and folic acid during pregnancy and the first 3 months postpartum and children received lipid-based nutrient supplements from 6 to 24 months of age; IFA-MNP, women received iron and folic acid during pregnancy and the first 3 months postpartum and children received micronutrient powder from 6 to 24 months of age; LNS-LNS, women and children received lipid-based nutrient supplements. “No show” refers to participants for whom data collection was not completed at that time point, primarily due to travel out of the study area or refusal because of illness or other reasons. [file 12887_2019_1738_MOESM2_ESM.pdf]
